# Experimental demonstration of classical analogous time-dependent superposition of states

**DOI:** 10.1038/s41598-022-27239-y

**Published:** 2022-12-30

**Authors:** Kazi T. Mahmood, M. Arif Hasan

**Affiliations:** Department of Mechanical Engineering, Wayne State University, Detroit, MI 48202 USA

**Keywords:** Applied physics, Information theory and computation

## Abstract

One of the quantum theory concepts on which quantum information processing stands is superposition. Here we provide experimental evidence for the existence of classical analogues to the coherent superposition of energy states, which is made possible by the Hertz-type nonlinearity of the granules together with the external driving field. The granules’ nonlinear vibrations are projected into the linear modes of vibration, which depend on one another through the phase and form a coherent superposition. We show that the amplitudes of the coherent states form the components of a state vector that spans a two-dimensional Hilbert space, and time enables the system to span its Hilbert space parametrically. Thus, the superposition of states can be exploited in two-state quantum-like computations without decoherence and wave function collapse. Finally, we demonstrate the experimental realization of applying a reversible Hadamard gate to a pure base state that brings the state into a superposition.

## Introduction

The increased demand for quantum information science (QIS) and quantum computing^1–5^ dictates a closer analysis of the topic and its methods. A quantum bit (qubit) is the essential component of QIS and a two-state quantum–mechanical system that can, most importantly, exist in superposition. A new, distinct state with specific quantitative connections to the first two given states is referred to as a superposition of the former two. In addition to providing superposition of states, the capacity to correlate between the subsystems through entanglement is what makes qubits so powerful for information processing. However, because of the environment’s fast ability to destroy the delicate coherence of these states, it is challenging to create and observe initially prepared quantum superposed states. As a result, particles and some microscopic objects that have been cooled to a temperature close to absolute zero^6–8^ thus exhibit such quantum superposition^9,10^. On the other hand, topological quantum computing (TPC), where the particle worldlines’ topological properties on a macroscopic scale are all that matter, uses non-Abelian forms of matter to store quantum information in an effort to construct a more robust qubit ^11,12^. However, according to Frolov’s commentary in Nature ^13^, the Majorana particle dispute is undermining the TPC field's trust because it is very challenging to create a topological qubit.. Consequently, the research of superpositions of other macroscopic states, or macroscopic superposition states, has been actively pursued over the past few decades and successfully experimentally demonstrated in a variety of systems, including trapped ions^14^, Bose–Einstein condensates^15,16^, and atomic systems^17^. Additionally, by driving the qubit monochromatically^18^ or by detecting the two-phonon interaction between a mechanical oscillator and a spin qubit^19^, the macroscopic quantum superposition in a qubit-oscillator system has also been explored. Very recently, Wood et al. suggested a platform to create macroscopic superpositions and a plan to place a 250 nm diameter diamond into a superposition in order to investigate the macroscopic boundaries of quantum mechanics^20^.

Additional perspectives for QIS and quantum mechanics applications and technological advancements are provided by the establishment of acoustic analogues of quantum phenomena^21^. One notable instance is the linear elastic field, which has been shown to theoretically and experimentally produce coherent superpositions of classical harmonic waves that are analogous to spin states in quantum mechanics^22^. Nevertheless, in order to observe true quantum-like phenomena, the mechanical system’s nonlinearity is necessary. The creation of mechanical non-Gaussian states with a negative Wigner function is one such example. It has been suggested that dissipation^23–26^, quantum tunneling with a double-well optomechanical potential^27,28^, periodic qubit flipping^29^, quantum interference effects^30^, optical field conditional measurement^31–33^, and modulated photon-hopping interaction between two cavities in an optomechanical system^34,35^ can produce macroscopic non-Gaussian superposition states. These methods are based on the nonlinear interaction between optical and mechanical degrees of freedom. In the same direction, in Ref.^36^, an experimental generation of the macroscopic superposition state was made possible by the Kerr-type nonlinearity by varying the driving field’s amplitude. To our knowledge, however, no comparable work has been done in nonlinear classical elastic systems where nonlinearity has been exploited to create a superposition of states. An elastic bit in a nonlinear classical system can create a superposition of states that is stable at ambient temperature and decoherence-free. Furthermore, since it represents an actual amplitude rather than a probability amplitude, it can be measured directly in the absence of wave function collapse. These characteristics make it possible for an elastic bit to be realized experimentally, providing a revolutionary new way to accomplish some of the objectives of quantum information technology utilizing materials-based quantum analogues. The present study’s objective is to experimentally demonstrate the possibility of preparing acoustic analogues of superposition states in a nonlinear acoustic granular medium and manipulating the superposition of Bloch states. More specifically, by harmonically driving a nonlinear system composed of two spherical granules, we experimentally demonstrate that the nonlinear normal modes can be expressed on a linear normal mode orthonormal basis with time-dependent amplitudes. These amplitudes form the components of a state vector that spans a two-dimensional (2D) Hilbert space parametrically with time. Thus, they serve as analogues of the qubit-like time-dependent coherent superpositions of states. In addition, we experimentally demonstrate that the frequency and amplitude of the external drivers applied to the nonlinear system are essential factors in navigating the elastic Bloch sphere. Most profoundly, since the system under consideration is nonlinear, we experimentally show that time permits the parametric exploration of the superposition of Bloch states.

## Results

### Experimental preparation and manipulation of classical coherent superpositions

To prepare and navigate classical analogous superposition of states using a nonlinear medium, we designed an experimental fixture consisting of a one-dimensional (1D) system of two homogeneous spherical elastic granules under external harmonic loading (details can be found in the “Methods” section). To minimize the error in the experimental results, the oscilloscope records the response signals and averages them over 256 times. Additionally, the soft plastic material employed in the vise jaw that holds the transducers reduces vibration transmission to the supporting experimental setups. Moreover, the masses and transducers’ center-to-center alignment was guaranteed with human-precision accuracy. Finally, we ensured the experiment was carried out over a short interval to avoid the couplant becoming contaminated with moisture and dust particles, which might impact the damping property.

**Figure 1:**
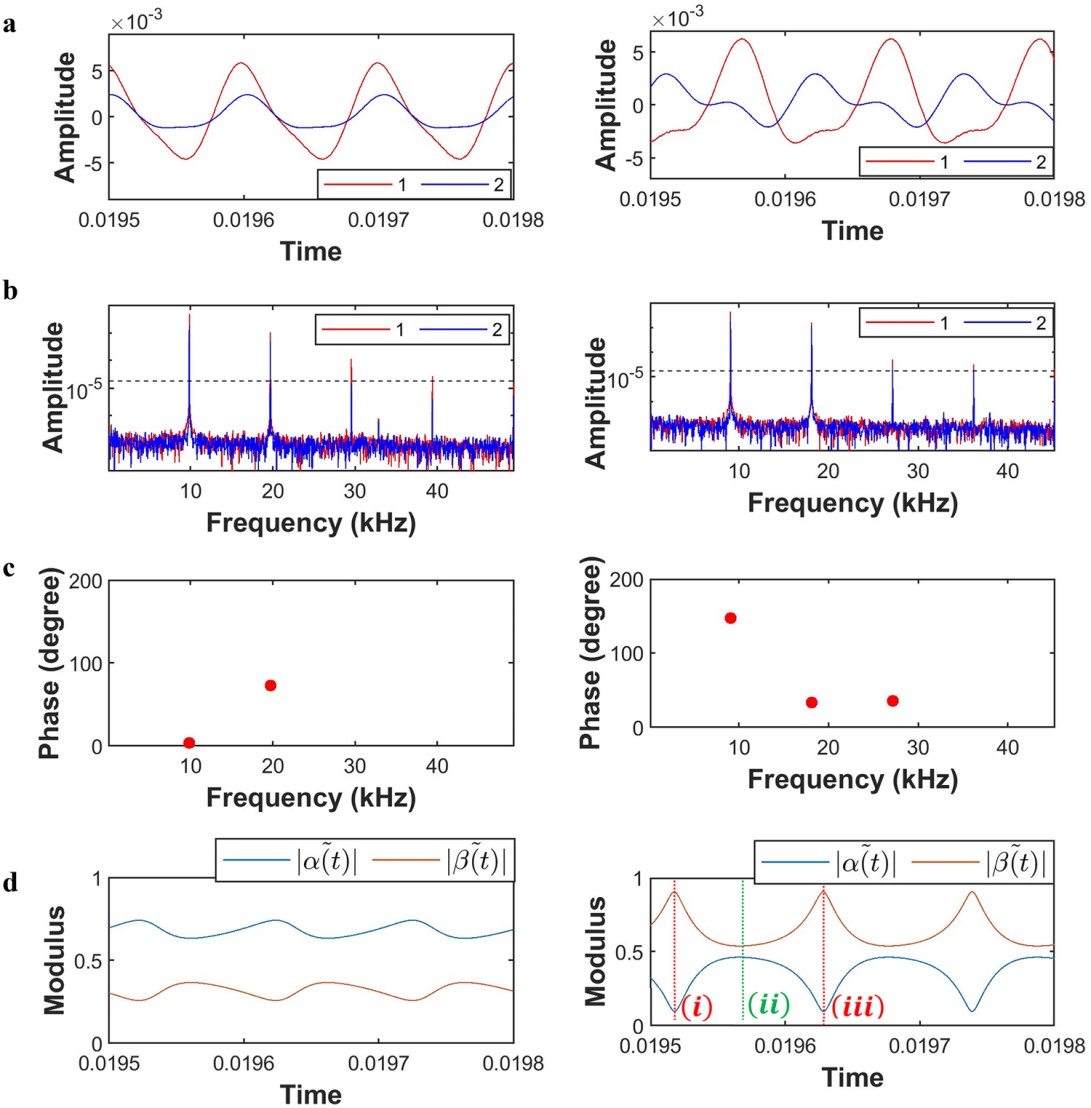
Measurement and tunability of classical analogue to superposition of states. **(a)** Amplitude versus time recorded by the detecting transducers of each granule at steady state, revealing rich nonlinear responses of the system. Left panel: driving frequency ${\omega }_{D}=9.85 \mathrm{kHz}$ and driving amplitude $100 {V}_{p-p}$; Right panel: driving frequency ${\omega }_{D}=9.05 \mathrm{kHz}$ and driving amplitude $100 {V}_{p-p}$. **(b)** Temporal Fourier transform of the granules amplitudes, and **(c)** phase differences between granules; revealing the combinations of ${E}_{1}$ and ${E}_{2}$ eigenstates associated with each characteristic frequency. In (b), the dashed horizontal line indicates the amplitude threshold for the selection of dominant characteristic frequencies. **(d)** Time evolution of the modules of the complex amplitudes, $\widetilde{\alpha }\left(t\right)$ and $\widetilde{\beta }\left(t\right)$, of two mutually orthogonal states $\left|{E}_{1}\right.\rangle$ and $\left|{E}_{2}\right.\rangle$. In (d), the vertical lines labeled (i), (ii), and (iii) correspond to three different time instants: $\left(\mathrm{i}\right)={t}_{1},\left(\mathrm{ii}\right)={t}_{2},\mathrm{and} \left(\mathrm{iii}\right)={t}_{3},$ where ${t}_{3}>{t}_{2}>{t}_{1}$.

To experimentally observe various nonlinear responses in such a damped-driven, essentially nonlinear granular system, we fix the amplitudes of external excitations by tuning peak-to-peak voltage. The frequency of the external driver (${\omega }_{D}$), however, varies from $100 \mathrm{Hz}$ to $20 \mathrm{kHz}$ with an increment of $10 \mathrm{Hz}$ with a resting period for obtaining a steady state. For different combinations of driving conditions (frequency and amplitude), we experimentally obtain Fig. 1a that shows the time series of the transmission amplitudes recorded by the detecting transducers of each granule at a steady state. The transmission amplitude field in Fig. 1a is the Fourier sum of the linear and nonlinear modes, each with its characteristic frequency. This is revealed in Fig. 1b through the temporal Fourier transform (fft) of the granule’s amplitude. To identify the dominant characteristic frequencies in the system, we set a threshold of 1% of the maximum amplitude to eliminate noise (dashed line in Fig. 1b). Moreover, we calculate the phase difference between granules for each dominant characteristic frequency. For example, in Fig. 1c left panel, we see that for the lowest dominant characteristic frequency of $\omega ={\omega }_{D}=9.85 \mathrm{kHz}$, corresponding to the driving frequency, the phase difference between granules is close to zero. This implies that at the characteristic frequency $\omega ={\omega }_{D}=9.85 \mathrm{kHz}$, the amplitude field of the granules’ system can be described by the state: $${E}_{1}=\frac{1}{\sqrt{2}}\left(\begin{array}{c}1\\ 1\end{array}\right)$$. On the contrary, if we change the driving frequency to ${\omega }_{D}=9.05 \mathrm{kHz};$ for such, at the lowest dominant characteristic frequency, $\omega ={\omega }_{D}=9.05 \mathrm{kHz}$, we observe in Fig. 1c right panel that the phase difference between granules is close to $\pi$. Hence, at this characteristic frequency, the amplitude field can be described by the state: $${E}_{2}=\frac{1}{\sqrt{2}}\left(\begin{array}{c}1\\ -1\end{array}\right)$$. Next, at the second and third higher harmonics, $2{\omega }_{D}$ and $3{\omega }_{D}$, we observe in Fig. 1c left and right panel that the phase difference between granules is neither 0 nor $\pi$, and hence the states can be described by a combination of ${E}_{1}$ and ${E}_{2}$.

The $${E}_{1}=\frac{1}{\sqrt{2}}\left(\begin{array}{c}1\\ 1\end{array}\right)$$ and $${E}_{2}=\frac{1}{\sqrt{2}}\left(\begin{array}{c}1\\ -1\end{array}\right)$$ states are the corresponding in-phase and out-of-phase eigenmodes of the linearized granular system (details are provided in the “Methods” section). It is emphasized that even though nonlinear modes do not possess orthogonality properties (as do the linear normal modes) ^37–39^, the combinations of ${E}_{1}$ and ${E}_{2}$ form a complete orthonormal basis for the two-mass granular system. Therefore, we can form a basis for the states of the granular system in the form of ${E}_{1}$ and ${E}_{2}$. In this basis, for any specific characteristic frequency, $\omega ,$ the amplitude field can be written as:

1 $$\left(\begin{array}{c}\left|{C}_{{u}_{1}}\right|{e}^{i{\varphi }_{{u}_{1}}}\\ \left|{C}_{{u}_{2}}\right|{e}^{i{\varphi }_{{u}_{2}}}\end{array}\right){e}^{i\omega t}\equiv \frac{1}{\sqrt{{\left|\alpha \right|}^{2}+{\left|\beta \right|}^{2}}}\left(\alpha {E}_{1}+\beta {E}_{2}\right){e}^{i\omega t}$$

Here, ${C}_{{u}_{1}},{C}_{{u}_{2}}$ are the amplitudes and ${\varphi }_{{u}_{1}},{\varphi }_{{u}_{2}}$ are the absolute phases of the granule amplitudes at the specific characteristic frequency $\omega$. Through the detecting transducers, ${C}_{{u}_{1}},{C}_{{u}_{2}},{\varphi }_{{u}_{1}},$ and ${\varphi }_{{u}_{2}}$ are experimentally measurable quantities (Fig. 1b,c). Here, $${E}_{1}=\frac{1}{\sqrt{2}}\left(\begin{array}{c}1\\ 1\end{array}\right)$$ and $${E}_{2}=\frac{1}{\sqrt{2}}\left(\begin{array}{c}1\\ -1\end{array}\right)$$ form a complete orthogonal basis for the system. Moreover, the amplitudes, $\alpha$ and $\beta$, are complex quantities, since simplifying Eq. (1), we find

2 $$\left(\begin{array}{c}\left|{C}_{{u}_{1}}\right|{e}^{i{\varphi }_{{u}_{1}}}\\ \left|{C}_{{u}_{2}}\right|{e}^{i{\varphi }_{{u}_{2}}}\end{array}\right){e}^{i\omega t}\equiv \frac{1}{\sqrt{2\left({\left|\alpha \right|}^{2}+{\left|\beta \right|}^{2}\right)}}\left(\begin{array}{c}\alpha +\beta \\ \alpha -\beta \end{array}\right){e}^{i\omega t}.$$

Using Eq. (2), we can calculate ${E}_{1}$ and ${E}_{2}$ states amplitude coefficients. Hence, similarly to a quantum system, after normalization Eq. (1), a unit vector can be used to describe the state of the granular system in a complex vector space, known as state space or Hilbert space. Moreover, the vectors ${E}_{1}$ and ${E}_{2}$ are two mutually orthogonal eigenstates of the system and therefore, form an orthonormal basis for a 2D Hilbert space. Therefore, using an analogy with a quantum system, we use the Dirac notation for vectors and apply it to the elastic states of the system by writing vectors in state space as:

3 $$\left(\begin{array}{c}\left|{C}_{{u}_{1}}\right|{e}^{i{\varphi }_{{u}_{1}}}\\ \left|{C}_{{u}_{2}}\right|{e}^{i{\varphi }_{{u}_{2}}}\end{array}\right){e}^{i\omega t}\equiv \frac{1}{\sqrt{{\left|\alpha \right|}^{2}+{\left|\beta \right|}^{2}}}\left(\left|\alpha \right|{e}^{i{\phi }_{\alpha }}\left|{E}_{1}\right.\rangle +\left|\beta \right|{e}^{i{\phi }_{\beta }}\left|{E}_{2}\right.\rangle \right){e}^{i\omega t},$$

where ${\phi }_{\alpha }={\mathrm{tan}}^{-1}\left(\frac{\eta \omega }{m{\omega }_{1}^{2}-{m\omega }^{2}}\right)$ and ${\phi }_{\beta }={\mathrm{tan}}^{-1}\left(\frac{\eta \omega }{m{\omega }_{2}^{2}-{m\omega }^{2}}\right);$ $m$ is the mass of the granule, $\eta$ is the system damping, and ${\omega }_{1}$ and ${\omega }_{2}$ are the eigen frequencies of the in-phase and out-of-phase modes of the eigen vectors of the linearized granular system (refer to the “Methods” section for details). The vibrations of the granules are represented by Eq. (3) projected into the two possible modes of vibration. The components of Eq. (3) are dependent on each other through the phase and form a coherent superposition of states in the space of two possible forms of vibration; since in-phase $\left({E}_{1}\right)$ and out-of-phase $\left({E}_{2}\right)$ vibration modes are physically distinguishable independent states. Moreover, the components of Eq. (3) physically correspond to superposed states, i.e., the characteristics of a pure in-phase eigenstate for ${\phi }_{\beta }-{\phi }_{A}=0$ and the characteristics of a pure out-of-phase eigenstate for ${\phi }_{\beta }-{\phi }_{A}=\pi$. In contrast to classical mixed states or classical nonseparable combinations of longitudinal and torsional/shear modes, the superposition of states of Eq. (3) is coherent through the phase. We defined coherent states as states that retain their superposition characteristic, such that, the ${E}_{1}$ and ${E}_{2}$ eigenstates have a constant phase and exhibit interference for a given time instant. Moreover, over time, the coherent state stays coherent, but its phase relation evolves in time. The superposition of states of Eq. (3) is also different than the problems that fall into a class that is nonseparable classically, where the nonseparability stems from media corners and crack edges^40^.

Hence, the total displacement amplitude field of the nonlinear granular system (Fig. 1a) can be written as the linear combination:

4 $$\overrightarrow{U}=\left(\begin{array}{c}{u}_{1}\\ {u}_{2}\end{array}\right)={\sum }_{n}\left(\begin{array}{c}\left|{C}_{1,n}\right|{e}^{i{\varphi }_{{u}_{1,n}}}\\ \left|{C}_{2,n}\right|{e}^{i{\varphi }_{{u}_{2,n}}}\end{array}\right){e}^{i{\omega }_{n}t}\equiv \frac{1}{\sqrt{{\left|\widetilde{\alpha }\left(t\right)\right|}^{2}+{\left|\widetilde{\beta }\left(t\right)\right|}^{2}}}\left[\widetilde{\alpha }\left(t\right)\left|{E}_{1}\right.\rangle +\widetilde{\beta }\left(t\right)\left|{E}_{2}\right.\rangle \right],$$

where $\widetilde{\alpha }\left(t\right)=\left({\sum }_{n}^{ }\frac{1}{\sqrt{{\left|{\alpha }_{n}\right|}^{2}+{\left|{\beta }_{n}\right|}^{2}}}{\alpha }_{n}{e}^{i{\omega }_{n}t}\right)$ and $\widetilde{\beta }\left(t\right)=\left({\sum }_{n}^{ }\frac{1}{\sqrt{{\left|{\alpha }_{n}\right|}^{2}+{\left|{\beta }_{n}\right|}^{2}}}{\beta }_{n}{e}^{i{\omega }_{n}t}\right)$. Here, ${u}_{1},{u}_{2}$ denote the displacements of the granules center from its equilibrium position. The total displacement field is therefore expanded on the basis of ${E}_{1}$ and ${E}_{2}$ with time-dependent complex coefficients, $\widetilde{\alpha }\left(t\right)\mathrm{ and }\widetilde{\beta }\left(t\right)$, where ${\alpha }_{n},{\beta }_{n};n=\mathrm{1,2},\dots$ are the $n$-th complex amplitudes of the $n$th dominant characteristic frequency identified in Fig. 1b for the mutually orthogonal eigenstates ${E}_{1}$ and ${E}_{2}$. On that basis, the modal contribution in the mode superposition of the total displacement field can be written in the form of a column displacement state vector, $|\psi \rangle$:

5 $$|\psi \rangle =\left(\begin{array}{c}{\psi }_{0}\\ {\psi }_{1}\end{array}\right)=\left(\begin{array}{c}{\sum }_{n}\frac{1}{\sqrt{{\left|{\alpha }_{n}\right|}^{2}+{\left|{\beta }_{n}\right|}^{2}}}{\alpha }_{n}{e}^{i{\omega }_{n}t}\\ {\sum }_{n}\frac{1}{\sqrt{{\left|{\alpha }_{n}\right|}^{2}+{\left|{\beta }_{n}\right|}^{2}}}{\beta }_{n}{e}^{i{\omega }_{n}t}\end{array}\right)=\left(\begin{array}{c}\widetilde{\alpha }\left(t\right)\\ \widetilde{\beta }\left(t\right)\end{array}\right).$$

This two-level subsystem represents an elastic bit and is isomorphic to a qubit. Here the coefficients of the superposition of states $\widetilde{\alpha }\left(t\right)$ and $\widetilde{\beta }\left(t\right)$ are time dependent. To demonstrate, let us focus on the specific driving condition and system parameters of Fig. 1b left panel. We see that by keeping the first two dominant characteristic frequencies, we find

6 $$\widetilde{\alpha }\left(t\right)=\left(\frac{1}{\left|{\alpha }_{1}\right|}{\alpha }_{1}{e}^{i{\omega }_{D}t}+\frac{1}{\sqrt{{\left|{\alpha }_{2}\right|}^{2}+{\left|{\beta }_{2}\right|}^{2}}}{\alpha }_{2}{e}^{i2{\omega }_{D}t}\right), \widetilde{\beta }\left(t\right)=\left(\frac{1}{\sqrt{{\left|{\alpha }_{2}\right|}^{2}+{\left|{\beta }_{2}\right|}^{2}}}{\beta }_{2}{e}^{i2{\omega }_{D}t}\right).$$

From the fft plot of Fig. 1b left panel, we see that the amplitude ${\alpha }_{1}$ of frequency $\omega$ is the dominant term, and it corresponds to pure $\left|{E}_{1}\right.\rangle$ eigenstate since at that frequency the phase difference is almost zero (cf. Figure 1c left panel). Hence, in Eq. (6), we expect that the coefficient $\widetilde{\alpha }\left(t\right)$ will be dominant in comparison to the coefficient $\widetilde{\beta }\left(t\right)$, as is also confirmed in Fig. 1d left panel. In Fig. 1d, we observe the time dependencies of the modules of the complex amplitudes, $\widetilde{\alpha }\left(t\right)$ and $\widetilde{\beta }\left(t\right)$, of two mutually orthogonal states $\left|{E}_{1}\right.\rangle$ and $\left|{E}_{2}\right.\rangle$. Next, if we move to a different driving frequency, we observe from Fig. 1d right panel that it is possible to vary the coefficients of the coherent superposition of states significantly. In Fig. 1d right panel, we see that for the case of driving frequency $\omega =9.05 \mathrm{kHz}$, the coefficient $\widetilde{\beta }\left(t\right)$ dominates, which can be inferred from the right panels of Fig. 1b,c since ${E}_{2}$ eigenstate dominates at the first dominant characteristic frequency. Hence, the elastic bit states live in a 2D Hilbert space, and through the driving parameters, we can navigate the Hilbert space significantly. An elastic bit is, therefore, a classical analogue with respect to superposition of a qubit-the critical component of quantum computing platforms.

## Experimental realization of Hadamard gate

As seen in Fig. 1d, time allows the system to tune the superposition of states created by the two eigenmodes. Hence, the passage of time is therefore equivalent to applying a unitary transformation to the superposed states. To illustrate this point, let us focus on time instant ${t}_{1}$ labeled (i) in Fig. 1d right panel. For such an instant, using Eq. (5), the modal contribution in the mode superposition of the displacement state vector can be written as:

$$|\psi \rangle =\left(\begin{array}{c}\frac{1}{\left|{\alpha }_{1}\right|}{\alpha }_{1}{e}^{i{\omega }_{D}{t}_{1}}+\frac{1}{\sqrt{{\left|{\alpha }_{2}\right|}^{2}+{\left|{\beta }_{2}\right|}^{2}}}{\alpha }_{2}{e}^{i2{\omega }_{D}{t}_{1}}+\frac{1}{\sqrt{{\left|{\alpha }_{3}\right|}^{2}+{\left|{\beta }_{3}\right|}^{2}}}{\alpha }_{3}{e}^{i3{\omega }_{D}{t}_{1}}\\ \frac{1}{\left|{\beta }_{1}\right|}{\beta }_{1}{e}^{i{\omega }_{D}{t}_{1}}+\frac{1}{\sqrt{{\left|{\alpha }_{2}\right|}^{2}+{\left|{\beta }_{2}\right|}^{2}}}{\beta }_{2}{e}^{i2{\omega }_{D}{t}_{1}}+\frac{1}{\sqrt{{\left|{\alpha }_{3}\right|}^{2}+{\left|{\beta }_{3}\right|}^{2}}}{\beta }_{3}{e}^{i3{\omega }_{D}{t}_{1}}\end{array}\right).$$

**Figure 2:**
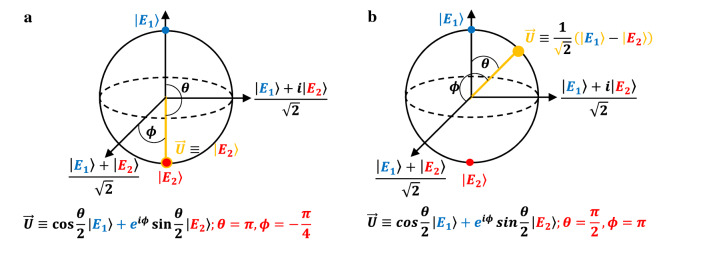
Classical analogue of Hadamard gate on Bloch sphere. The Hadamard gate ‘rotates’ the initial pure state of $\left|{E}_{2}\right.\rangle$ **(a)** (also labeled (i) in Fig. 1d right panel) to a superposition of states **(b)** (also labeled (ii) in Fig. 1d right panel) through a unitary transformation $$\frac{1}{\sqrt{2}}\left(\begin{array}{cc}1& 1\\ 1& -1\end{array}\right)$$.

However, in the above equation, ${\alpha }_{1}\approx 0$, since the phase difference between granules is close to $\pi$ at the lowest dominant characteristic frequency ${\omega }_{D}$, as illustrated in Fig. 1c right panel. Hence, at this characteristic frequency, the amplitude field can be described by the state: $${E}_{2}=\frac{1}{\sqrt{2}}\left(\begin{array}{c}1\\ -1\end{array}\right)$$. Therefore, the superposition of states can be written as:

7 $$\overrightarrow{U}\equiv \left(\begin{array}{c}0.0007-0.0193i\\ 0.7565-0.6538i\end{array}\right)\left(\left|{E}_{1}\right.\rangle +\left|{E}_{2}\right.\rangle \right)\equiv \left(0.0194{e}^{-i\frac{\pi }{2}}\right)\left|{E}_{1}\right.\rangle +\left(0.9998{e}^{-i\frac{\pi }{4}}\right)\left|{E}_{2}\right.\rangle \equiv \mathrm{cos}\frac{\theta }{2}\left|{E}_{1}\right.\rangle +{e}^{i\phi }\mathrm{sin}\frac{\theta }{2}\left|{E}_{2}\right.\rangle ;\theta =\pi ,\phi =-\frac{\pi }{4},$$

with an estimated uncertainty of $\frac{\pi }{20}$ in $\theta$ and $\frac{3\pi }{50}$ in $\phi$, which depends on the amplitude threshold value used for selecting dominant characteristic frequencies (cf. Figure 1b). The state corresponds to time instant ${t}_{1}$ of Fig. 1d right panel (Eq. (7)) is also depicted in Fig. 2a on a Bloch sphere. A Bloch sphere is a useful tool for visualizing superposition states. A linear combination of the $\left|{E}_{1}\right.\rangle$ and $\left|{E}_{2}\right.\rangle$ states with complex coefficients is represented by a point on this sphere (Fig. 2a). Similarly, the state corresponds to time instant ${t}_{2}$ of Fig. 1d right panel (labeled (ii)) can also be written as:

8 $$\overrightarrow{U}\equiv \left(\begin{array}{c}0.5354-0.476i\\ -0.5344+0.4486i\end{array}\right)\left(\left|{E}_{1}\right.\rangle +\left|{E}_{2}\right.\rangle \right)\equiv \left(0.716{e}^{-i\frac{\pi }{4}}\right)\left|{E}_{1}\right.\rangle +\left(0.698{e}^{i\frac{3\pi }{4}}\right)\left|{E}_{2}\right.\rangle \equiv \mathit{cos}\frac{\theta }{2}\left|{E}_{1}\right.\rangle +{e}^{i\phi }\mathit{sin}\frac{\theta }{2}\left|{E}_{2}\right.\rangle ;\theta =\frac{\pi }{2},\phi =\pi ,$$

and the state is again represented in Fig. 2b on a Bloch sphere. A Hadamard gate ‘rotates’ the initial state of Eq. (7) (labeled (i)), which is almost a pure state $\left|{E}_{2}\right.\rangle$, to a superposition state of the form Eq. (8) (labeled (ii)) through the transformation:

9 $$\frac{1}{\sqrt{2}}\left(\begin{array}{cc}1& 1\\ 1& -1\end{array}\right)\left(\begin{array}{c}0.007-0.0193i\\ 0.7565-0.6538i\end{array}\right)=\left(\begin{array}{c}0.5354-0.476i\\ -0.5344+0.4486i\end{array}\right)$$

The transformation matrix $$\frac{1}{\sqrt{2}}\left(\begin{array}{cc}1& 1\\ 1& -1\end{array}\right)$$ is the usual Hadamard gate^41^. If we apply the Hadamard gate on the state (ii), we return to the initial pure state $\left|{E}_{2}\right.\rangle$ at time instant ${t}_{3}$ (labeled (iii)).

## Discussion

We have demonstrated the preparation and manipulation of classical analogous to a quantum superposition of Bloch states via a controllable, essentially nonlinear granular system. In the current setup, a Hertz-type nonlinear interaction among granules is induced by harmonically driving the granular system. By expressing the modes of vibration of the granules on a linear normal mode orthonormal basis, we have shown that we can create a classical wave function that consists of a superposition of energy states. In addition, the amplitudes of the coherent states are complex. One notable feature of the current study is that since classical wave functions are amplitudes, they do not collapse upon measurement like quantum wave functions (probability amplitude). We have explored different ways of manipulating the complex amplitude coefficients of these coherent superpositions of states by varying the external driver’s parameters. Since the granular system under consideration is nonlinear, we have observed that not only the frequency of the external driver is an essential parameter for navigating the complex amplitudes of the superposition of states but also the amplitude of the external driver. One distinguishing and most crucial feature of the current study compared to work^42^ in a linear system is that in the previous study, the authors showed that the modulus of the complex amplitudes of the superposition of states could only be tuned by varying the amplitudes of the external driver. To adjust the phase of the complex amplitudes of the superposition of states, the authors changed the phase of the external driver^42^. In contrast, in the current work, we have revealed that since the granular system is an essentially nonlinear system, by tuning either the frequency or amplitude of the external driver, it is possible to tune both the modulus and the phase of the coherent superposition of states. Hence, nonlinear elasticity is a potential design parameter in extending the range of the elastic superposition of states that can be explored via an external driver. Moreover, we have also observed that the coefficients of the complex amplitudes are time dependent. When the external driver is used to prepare the two orthogonal states of the nonlinear granular system into a coherent superposition, they are in a state of superposition with the time evolution. After a full period, the superposition states disappear, and the mechanical modes become pure eigenstates.

We have demonstrated how to manipulate the coherent states in time without tuning the external driver’s amplitudes and frequency, which is equivalent to applying gate operations. In particular, we have focused on the Hadamard gate, a single qubit operation in quantum computing. Quantum gates on a Josephson junction qubit are carried out by electromagnetic impulses sent to the qubits at microwave frequencies ^43^. On the contrary, in our nonlinear classical system, time permits the parametric exploration of the superposition states, i.e., the external driver’s frequency, amplitude, and a particular duration determine the angle of rotation of the superposition of states around a particular axis of the Bloch sphere. The prepared and transformed superpositions of states are classical in nature and allow for experimental analysis (preparation, manipulation, and observation) without the additional quantum gate operations required in a true quantum algorithm. One can, for instance, operate on stable, non-decoherent, directly measurable, coherent superpositions of states of the elastic bit system without the wave function collapse. In a true quantum algorithm, however, the logic gates act on a superposition of qubit states to produce a predictable output state. Due to the probabilistic nature of the quantum wave function, many measurements are therefore required to determine a quantum superposition of states.

Finally, using a two-granule system, the current paper focuses on a single two-level elastic bit analogous to a single qubit. Moreover, it has been theoretically demonstrated that depending on the different ordered arrangements of the granules, the three-granule system supports four nonlinear normal modes (NNMs), and the four-granule system supports eight NNMs ^44,45^. Hence, it is possible to extend this work to create analogous multi-level quantum states such as qudits. Further, coupling multiple elastic bits through classical entanglement or, more precisely, through non-separability is essential for implementing information processing platforms that can take on the exponential complexity associated with the non-separability of states in coupled systems. In this direction, additional future work will investigate the possibility of achieving non-separability between different possible degrees of freedom in a coupled granular system. For instance, in^46^, we have shown that the modes of a coupled granular network can be decomposed across and along the network, forming an orthonormal basis for two two-dimensional Hilbert spaces. This is analogous to two qubits; thus, creating two-qubit Controlled NOT-type gates will be possible. It is true that in such a setting, time will permit the parametric exploration of the superposition of Bloch states. Therefore, creating a sequence of single or two-qubit analogue gates might be challenging because, as described by^47^, cascading two unitary transformations in a quantum harmonic oscillator produces a new transform with unrelated eigenvalues. However, in a coupled granule system, the coupling can be easily manipulated and tailored through choices of materials and fabrication to create strong correlations between the subsystems. Hence, we can create operations that can be carried out without breaking them down into a series of smaller steps.

## Methods

### Design of the experimental setup and theoretical model

**Figure 3:**
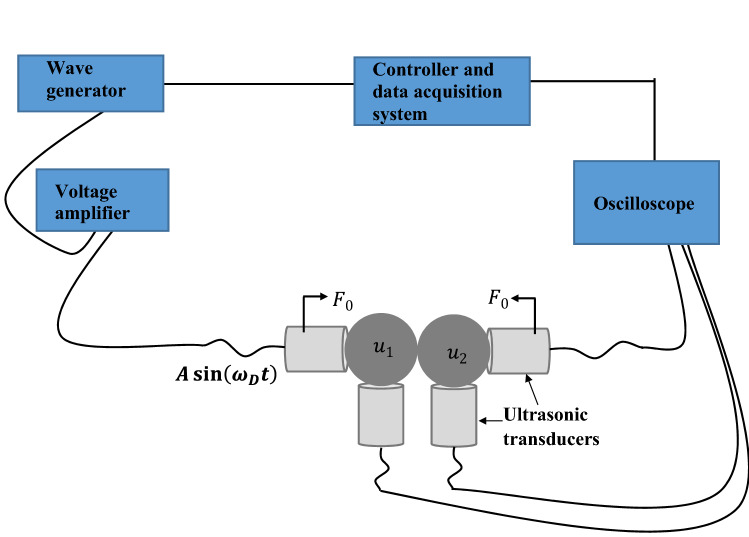
Experimental realization of time-dependent superposition of states. Schematic illustration of the experimental instrumentation used for the two-granule nonlinear system. The system is longitudinally driven by a single transducer at one end, and a set of transducers are utilized to detect the longitudinal modes of the granules.

The schematic illustration to experimentally realize classical time-dependent superposition of states is depicted in Fig. 3. Here, we seek to experimentally explore the response of two contacting granules (304 Stainless Steel: McMaster-Carr 9291K54, $1/2$ inch diameter, Young’s modulus $193 \mathrm{GPa}$, and density $7958$ $\mathrm{kg}/{\mathrm{m}}^{3}$) that are initially in contact with each other. A single transducer (V133-RM—Olympus IMS) drives the system at one end. Through PD200 amplifiers (PD200 is a high bandwidth, low-noise linear amplifier), the driving transducer is coupled to a waveform generator (B&K Precision 4055B). The waveform generator is set to vary the driving frequency in the system in a fixed excitation amplitude. The transducers and granules are connected center to center to detect the response in perfect alignment. To measure the signal generated in the system, the three recording transducers are connected to a Tektronix oscilloscope (MDO3024) and averaged across 256 time series, resulting in the response signals. Both the waveform generators and the oscilloscopes are connected to digital computers so that the experiments can be controlled, and the data can be processed. This is done with a custom algorithm created and implemented in the programming language MatLab. The experimental setup is devised to explore only longitudinal modes in the system. By limiting the translational motion of the granular system, it was experimentally feasible to neglect the rotating degrees of freedom of the granules due to their small relative displacement. Through the transducers, we are able to measure the amplitude field of the granules in the transverse direction, which maps the elastic field of longitudinal modes. The use of D12 ultrasonic couplant (gel type from Olympus-IMS) in conjunction with the longitudinal wave transducers suppress all nonlongitudinal modes (torsional, transversal, etc.) of the granules. Uniform compression force, ${F}_{0}$, is provided to both ends of the system using a bench vise to fix the initial displacement ${\delta }_{0}$ between the granules centers (Fig. 3).

Accordingly, the general mathematical expression of the nonlinear granular system (Fig. 3), restricted to 1D, reads:

10 $$\begin{aligned}& m{\ddot{u}}_{1}={k}_{NL}{\left[A\mathrm{sin}\left({\omega }_{D}t\right)-{u}_{1}+{\delta }_{0}\right]}_{+}^{3/2}-{k}_{NL}{\left({u}_{1}-{u}_{2}+{\delta }_{0}\right)}_{+}^{3/2}+\eta \left[{A}_{1}\omega \mathrm{cos}\left(\omega t\right)-{\dot{u}}_{1}\right] H\left(A\mathrm{sin}\left({\omega }_{D}t\right)-{u}_{1}+{\delta }_{0}\right)-\eta \left({\dot{u}}_{1}-{\dot{u}}_{2}\right)H\left({u}_{1}-{u}_{2}+{\delta }_{0}\right) \\ &m{\ddot{u}}_{2}={k}_{NL}{\left[{u}_{1}-{u}_{2}+{\delta }_{0}\right]}_{+}^{3/2}-{k}_{NL}{\left({u}_{2}+{\delta }_{0}\right)}_{+}^{3/2}+\eta \left[{\dot{u}}_{1}-{\dot{u}}_{2}\right] H\left({u}_{1}-{u}_{2}+{\delta }_{0}\right)-\eta \left({\dot{u}}_{2}\right)H\left({u}_{2}+{\delta }_{0}\right)\end{aligned}$$

In the theoretical model (10), we neglect the effects of gravity; however, the dissipative effect is considered since dissipation is an integral part of any physical system. In Eq. (10), ${\left(\alpha \right)}_{+}=\alpha\,\, \mathrm{for} \,\,\alpha \ge 0$ and ${\left(\alpha \right)}_{+}=0 \,\,\mathrm{for}\,\, \alpha <0$ and $H\left(\cdot \right)$ is the Heaviside function. The static Hertz law, which assumes that the characteristic time scale of granule-to-granule Hertzian interaction under compression is significantly higher than the characteristic time scale of elastic stress wave propagation inside a granule, has been experimentally verified for dynamic problems of spherical granules ^48^. Here, ${k}_{NL}=\frac{E\sqrt{2R}}{3\left(1-{\nu }^{2}\right)}$, $m=\frac{4}{3}\pi {R}^{3}\rho$ is the mass of the granules, $R$ is the radius, and $\rho$ is the density of granule material. The damping coefficient $\eta$ models the dissipation during Hertzian interactions between adjacent granules. The static overlap ${\delta }_{0}$ due to the applied static load simulates the applied pre-compression of the system scaled by the common granules’ radius, and the base periodic excitation $A\mathrm{sin}\left({\omega }_{D}t\right)$ is applied to the first mass of the system to model the excitation delivered by the excitation transducer.

If we perform a power series expansion of the forces of Eq. (10), and in the case of dynamical displacements significantly smaller in amplitude than the static overlap ${\delta }_{0}$, i.e., $\frac{\left|\Delta u\right|}{{\delta }_{0}}\ll 1$, where $\Delta u={u}_{1}-{u}_{2}$ or $\Delta u=A\mathrm{sin}\left({\omega }_{D}t\right)-{u}_{1}$ or $\Delta u={u}_{2}$, in Eq. (10), only the harmonic term of the expansion is retained. For such, the granular system can be considered as a linear lattice, and the equations of motion are reduced to^49,50^:

$$m{\ddot{u}}_{1}={k}_{L}\left[A\mathrm{sin}\left({\omega }_{D}t\right)-{u}_{1}\right]-{k}_{L}\left({u}_{1}-{u}_{2}\right)+\eta \left[{A}_{1}\omega \mathrm{cos}\left({\omega }_{D}t\right)-{\dot{u}}_{1}\right] -\eta \left({\dot{u}}_{1}-{\dot{u}}_{2}\right)$$

11 $$m{\ddot{u}}_{2}={k}_{L}\left({u}_{1}-{u}_{2}\right)-{k}_{L}\left({u}_{2}\right)+\eta \left({\dot{u}}_{1}-{\dot{u}}_{2}\right) -\eta \left({\dot{u}}_{2}\right)$$

Here, ${k}_{L}=\frac{3}{2}{k}_{NL}{\delta }_{0}^{1/2}$ is the equivalent linear spring constant. The sound velocity of such a 1D monoatomic granular system has been confirmed experimentally ^51^. Since the coupling stiffness of Eq. (11) is linear, the eigenstates of the linearized granular system should be $${E}_{1}=\frac{1}{\sqrt{2}}\left(\begin{array}{c}1\\ 1\end{array}\right)$$ and $${E}_{2}=\frac{1}{\sqrt{2}}\left(\begin{array}{c}1\\ -1\end{array}\right)$$; the corresponding in-phase and out-of-phase modes. These orthogonal states are mutually exclusive. As a result, for a linearized granular system with the presence of an external driver, we can write the displacement field similarly to Eq. (3) as follows:

12 $$\overrightarrow{U}=\left(\begin{array}{c}{u}_{1}\\ {u}_{2}\end{array}\right)=\left(\begin{array}{c}\left|{C}_{{u}_{1}}\right|{e}^{i{\varphi }_{{u}_{1}}}\\ \left|{C}_{{u}_{2}}\right|{e}^{i{\varphi }_{{u}_{2}}}\end{array}\right){e}^{i\omega t}\equiv \frac{1}{\sqrt{{\left|\alpha \right|}^{2}+{\left|\beta \right|}^{2}}}\left(\alpha \left|{E}_{1}\right.\rangle +\beta \left|{E}_{2}\right.\rangle \right){e}^{i\omega t}$$

For a linearized granular system, the amplitudes, $\alpha$ and $\beta$, can theoretically be estimated as $$\alpha =\frac{\frac{1}{\sqrt{2}}\left(\begin{array}{c}1\\ 1\end{array}\right).\left(\begin{array}{c}A\\ 0\end{array}\right)}{m{\omega }_{1}^{2}-\mathrm{m}{\omega }^{2}-i\eta \omega }$$ and $$\beta =\frac{\frac{1}{\sqrt{2}}\left(\begin{array}{c}1\\ -1\end{array}\right).\left(\begin{array}{c}A\\ 0\end{array}\right)}{m{\omega }_{2}^{2}-{m\omega }^{2}-i\eta \omega }$$, where ${\omega }_{1}$ and ${\omega }_{2}$ are the eigen frequencies of the in-phase and out-of-phase modes of the eigen vectors of the linearized granular system. Hence, through an external driver, it is possible to create a two-level acoustic analogue of a qubit. These coherent states are however time independent as time does not explicitly affects the amplitudes of each coherent states.

If the granular system is weakly compressed and if the granule displacements are either comparable or greater than the initial relative displacement ${\delta }_{0}$ arising from the static compression, then a very intriguing wave behavior develops for such a nonlinear regime. In 1D monoatomic granular crystals, this dynamical domain has received the majority of research attention^49,52,53^. In particular, in Refs  ^44,45,54,55^. the authors demonstrated that such nonlinear systems possess standing modes (referred to as nonlinear normal modes), and numerous subharmonic responses satisfying general $m:n$ rational granule frequency relationships. Note that in these studies, the authors have focused on a conservative system. In practice, dissipation needs to be considered.

## Data Availability

The data that support our findings of the present study are available from the corresponding author upon reasonable request.
